# The aqueous root extract of *Withania somnifera* ameliorates LPS-induced inflammatory changes in the *in vitro* cell-based and mice models of inflammation

**DOI:** 10.3389/fphar.2023.1139654

**Published:** 2023-06-12

**Authors:** Phulwanti Kumari Sharma, Lokesh Kumar, Yamini Goswami, Mukta Pujani, Madhu Dikshit, Ruchi Tandon

**Affiliations:** ^1^ Translational Health Science and Technology Institute, Faridabad, India; ^2^ ESIC Medical College and Hospital, Faridabad, India; ^3^ Pharmacology Division, Central Drug Research Institute, Lucknow, India

**Keywords:** *Withania somnifera*, cytokines, lung inflammation, PBMCs, TLR-4, LPS, COVID-19

## Abstract

**Introduction:** Most critically ill COVID-19 patients have bronchitis, pneumonia, and acute respiratory distress syndrome (ARDS) due to excessive inflammatory conditions. Corticosteroids have largely been prescribed for the management of inflammation in these patients. However, long-term use of corticosteroids in patients with comorbidities such as metabolic, cardiovascular, and other inflammatory disorders is ideally not recommended due to safety issues. A potential and safer anti-inflammatory therapy is therefore the need of the hour. *Withania somnifera* (WS), a well-known herbal medicine used during the pandemic in India to prevent SARS-CoV2 infection, also possesses anti-inflammatory properties.

**Methods:** In the present study, we, therefore, evaluated the effect of the aqueous extract of the roots of *W. somnifera* in the cell-based assays and in the experimental animal models of LPS-induced inflammation.

**Results:** In the NCI-H460, A549 cells and human peripheral blood mononuclear cells (PBMCs) pre-treatment with *W. somnifera* reduced the LPS-induced expression of the pro-inflammatory cytokines. In addition, *W. somnifera* extract also showed potent anti-inflammatory activity in the lung tissues of BALB/c mice challenged intranasally with LPS. We observed a marked reduction in the neutrophil counts in the broncho-alveolar lavage (BAL) fluid, inflammatory cytokines, and fibrosis in the mice lungs pre-treated with *W. somnifera*. Results obtained thus suggest the potential utility of *W. somnifera* extract in reducing airway inflammation and recommend the clinical evaluation of *W. somnifera* extract in COVID-19 patients with a high propensity for lung inflammation.

## Introduction

Coronavirus has got an overwhelming reach in the last 3 years among large populations and has a life-threatening impact on people with existing health issues such as respiratory, cardiac, and metabolic complications (Sanyaolu et al., 2020; Zheng et al., 2020) and those who survived have also been found to have a high prevalence of aberrant airway injuries including pneumonia, bronchitis and acute respiratory distress syndrome (George et al., 2020; Zhou et al., 2020; Allgood et al., 2021).

The hyper-inflammation in Corona-infected patients is a result of cytokine storm due to the excessive production of pro-inflammatory cytokines such as tumor necrosis factor-alpha (TNF-α), interleukin-6 (IL-6), interleukin-8 (IL-8), etc., leading to severe organ damage and may even lead to fatalities in severe cases (Mortaz et al., 2020; Tang et al., 2020; Zhou et al., 2020). The world health organization (WHO) recently released its updated guideline on 16 September 2022, and recommendations for the management of Covid associated complications (Sterne and Murthy, 2020; WHO, 2022). These recommendations suggest the use of remdesivir in combination with corticosteroids, interleukin-6 (IL-6) receptor blockers, and Janus kinase (JAK) inhibitors in patients with severe or critical COVID-19. This guideline has also modified the previous recommendations for the use of neutralizing monoclonal antibodies sotrovimab and casirivimab-imdevimab in patients with non-severe COVID-19.

In order to understand the mechanism of cytokine storm in Corona patients, we found that Corona virus-induced inflammatory profile seems to be similar to the inflammatory trigger induced by Toll-like Receptor 4 (TLR-4) activation. Lipopolysaccharide (LPS), an outer membrane constituent of gram-negative bacteria is a TLR-4 ligand that leads to an increase in the production of pro-inflammatory cytokines such as Tumor necrosis factor alpha (TNF-α), IL-6, and various other cytokines and chemokines in the cell-based models and also the in the mouse models (Park et al., 2004). Several anti-inflammatory drugs and herbal medications have been tried previously to inhibit LPS-induced inflammation in cell-based and mouse models (Yunhe et al., 2012; Huang et al., 2020).

Corticosteroids, as mentioned above, are the choice of medication by a standard medical practitioner to combat inflammation occurring in the lungs and other vital organs of patients infected with Coronavirus due to their potent anti-inflammatory properties (Li et al., 2021). Dexamethasone, a corticosteroid that has been previously shown to inhibit LPS-induced inflammation in both *in vitro* and *in vivo* models have been recommended for use in Corona patients also (RECOVERY Collaborative Group, 2021; Crothers et al., 2021). The unguided use of corticosteroids in patients, however, can be harmful in patients with metabolic diseases such as diabetes, hypertension, and other inflammatory diseases due to their side effects (Sterne and Murthy, 2020). To develop a safer approach for developing therapeutics for Corona patients, WHO has recommended a combination of herbal and modern systems of medicines to achieve the desired pharmacological response with minimal side effects which have also been recommended by the National Board of Medicinal Plants (NBMP) (2021), and Ministry of Ayush, Government of India (ebook, 20 medicinal plants 2021). Ayurveda, which is one of the oldest systems of medicine in the world, offers treatments based on combining products mainly derived from plants and natural resources from ancient Indian medical systems and offers such remedies which can be explored for the management of COVID-19 complications. In addition, several other complementary and traditional medicines have shown potential advantages for the management of various health issues in other countries also (Patwardhan et al., 2004; Ghasemian et al., 2016; Ni, 2020; Patwardhan et al., 2020; Shankar, 2020).

Recent study from this lab have demonstrated the protective effect of the aqueous extract of the *Withania somnifera* (WS) roots in the experimental models of SARS-CoV-2, T cell differentiation, and neutrophil functions (Rizvi et al., 2023) by reducing viral load and inflammation. While the therapeutic potential of Withaferin A (WA) and its derivatives has also been demonstrated in lung cancer. In the lung cancer cells (A549 and H1299), WA pre-treatment suppressed cell adhesion, migration, and invasion by downregulating the expression of tumor growth factor beta 1 (TGFβ1) and tumor necrosis factor *α* (TNFα) expression as well as epithelial-mesenchymal transition (Behl et al., 2020). Similar effects have also been reported in macrophage cell lines (THP-1 and RAW 246.7 cells). In yet another study, WA ameliorated *in vitro* and *in vivo* pulmonary fibrosis by modulating the interplay of fibrotic and matricellular proteins (Bale et al., 2018). Withanolides present in the aqueous extract of WS root inhibited TLR-4 activated innate inflammatory signal utilizing *in silico* and experimental approaches (Purushotham et al., 2017). In a recent study, WA and Withanones were found to inhibit SARS-CoV-2 proteins (Chakraborty et al., 2022). Since lung inflammation is one of the most common concerns in COVID-19 patients, we wanted to test whether WS extract has the potential to inhibit LPS-induced inflammation in the lung-derived cell lines and in the mice model of acute lung inflammation in a TLR-4-dependent manner.

For the *in vitro* studies, we used LPS-stimulated NCI-H460 and A549, lung-derived cell lines as well as human peripheral blood mononuclear cells (PBMCs) to further assess the anti-inflammatory potential of WS. In addition, we also assessed the anti-inflammatory potential of WS in BALB/c mice challenged with LPS by the intranasal route, which led to an increase in the migration of neutrophils in the broncho-alveolar lavage (BAL) fluid and induction of pro-inflammatory cytokines in the lung tissues. Our study demonstrates the potential use of the aqueous extract of WS in combating lung inflammation which can be evaluated further in the planned clinical setups.

## Material and methods

### Experimental animals

All the experiments were performed as per the guidelines of the Institutional Animal Ethics Committee (IAEC-THSTI/163) of the Translational Health Science and Technology Institute (THSTI), Faridabad, India, and all the experiments were carried out following the control and supervision of experiments on animals (CPCSEA) guidelines (Govt. of India).

8–12 weeks old female BALB/c mice (18–25 g) were procured from the Small Animal Facility (SAF) of Translational Health Science and Technology Institute (THSTI), Faridabad, and housed in individually ventilated cages with controlled air flow as per the institute’s experimental animal guidelines. Access to normal chow and water was provided to them *ad libitum*. The animals were acclimatized for 1 week in the above conditions prior to initiating the experiments.

### Cell culture

NCI-H460 cell line (human lung carcinoma cells) was procured from the National Centre of Cell Science, Pune, India, and was routinely maintained in RPMI-1640 medium containing 10% (v/v) fetal bovine serum (FBS), 100 units/ml penicillin and 100 μg/ml streptomycin (Invitrogen, United States). Cells were maintained in a humidified incubator with 5% CO_2_ at 37°C. A549 cells were procured from ATCC and maintained in DMEM containing 10% (v/v) fetal bovine serum (FBS), 100 units/ml penicillin and 100 μg/ml streptomycin (Invitrogen, United States) and maintained in a humidified incubator with 5% CO_2_ at 37°C, as above.

Aqueous extract of the *W. somnifera* (WS) roots was prepared in the good manufacturing practice (GMP) facility and was provided by NMPB, and its characterization has been reported previously (Kasarla et al., 2022).

### Quantitative real-time PCR (qPCR)

Total RNA was isolated from cells treated with different concentrations of WS and lungs using Trizol Reagent (Thermo Fisher Scientific), according to the manufacturer’s protocol and used for the cDNA synthesis. cDNA was synthesized with an iScript cDNA synthesis kit (Applied Biosystems, United States) as per the manufacturer’s protocol. The gene-specific primers (Table 1) were designed using NCBI primer blast tool, and qPCR was performed on the QuantStudio 6 (Applied Biosystems, United States) real-time PCR systems. Reactions were carried out in 10 μl volumes containing: 5 μl Sybr green Master Mix (PowerUp SYBR Green mix, Applied Biosystems; Cat. No. A25742), 0.5 μl forward and 0.5 μl reverse primers, 3 μl water, and 1 μl cDNA. Cycling program used was: 7 min at 95°C and then 40 cycles with 10 s at 95°C and 20 s at 60°C. The specificity of the amplicons was analysed by a thermal dissociation curve. Data were normalized against the housekeeping gene, 18S, or GAPDH.

**TABLE 1:** List of primers.

Gene	Forward primer	Reverse primer
Human primers		
TNF-alpha	CTC​TTC​TGC​CTG​CTG​CAC​TTT​G	ATG​GGC​TAC​AGG​CTT​GTC​ACT​C
IL-6	AGA​CAG​CCA​CTC​ACC​TCT​TCA​G	TTC​TGC​CAG​TGC​CTC​TTT​GCT​G
IL-1beta	CCA​CAG​ACC​TTC​CAG​GAG​AAT​G	GTG​CAG​TTC​AGT​GAT​CGT​ACA​GG
IL-8	GAG​AGT​GAT​TGA​GAG​TGG​ACC​AC	CAC​AAC​CCT​CTG​CAC​CCA​GTT​T
IL-18	GAT​AGC​CAG​CCT​AGA​GGT​ATG​G	CCT​TGA​TGT​TAT​CAG​GAG​GAT​TCA
IL-4	CCG​TAA​CAG​ACA​TCT​TTG​CTG​CC	GAG​TGT​CCT​TCT​CAT​GGT​GGC​T
CCL2	AGA​ATC​ACC​AGC​AGC​AAG​TGT​CC	TCC​TGA​ACC​CAC​TTC​TGC​TTG​G
18S	ACC​CGT​TGA​ACC​CCA​TTC​GTG​A	GCC​TCA​CTA​AAC​CAT​CCA​ATC​GG
Mouse primers		
Tnf-alpha	GGT​GCC​TAT​GTC​TCA​GCC​TCT​T	GCC​ATA​GAA​CTG​ATG​AGA​GGG​AG
Il-6	TAC​CAC​TTC​ACA​AGT​CGG​AGG​C	CTG​CAA​GTG​CAT​CAT​CGT​TGT​TC
Il-1beta	TGG​ACC​TTC​CAG​GAT​GAG​GAC​A	GTT​CAT​CTC​GGA​GCC​TGT​AGT​G
Il-18	GAC​AGC​CTG​TGT​TCG​AGG​ATA​TG	TGT​TCT​TAC​AGG​AGA​GGG​TAG​AC
Cxcl1	TCC​AGA​GCT​TGA​AGG​TGT​TGC​C	AAC​CAA​GGG​AGC​TTC​AGG​GTC​A
Il-4	ATC​ATC​GGC​ATT​TTG​AAC​GAG​GTC	ACC​TTG​GAA​GCC​CTA​CAG​ACG​A
Ccl2	GCT​ACA​AGA​GGA​TCA​CCA​GCA​G	GTC​TGG​ACC​CAT​TCC​TTC​TTG​G
18S	GCA​ATT​ATT​CCC​CAT​GAA​CG	GGC​CTC​ACT​AAA​CCA​TCC​AA

### *In vitro* anti-inflammatory activity of *Withania somnifera* and cell viability assay

NCI-H460 cells were seeded at a density of 1 ×10^4^ cells/well in RPMI medium with 10% FBS in a 96-well plate and incubated at 37°C in a CO_2_ incubator for 24 h followed by treatment with WS at 10, 3, 1, 0.3, 0.1, and 0.003 mg/ml. LPS (1 μg/ml) was added 30 min after the treatment with WS and the plate was incubated further in the CO_2_ incubator for 24 h. Dexamethasone was used as a positive control (10 µM). For gene expression analysis, cells were grown at a density of 4 × 10^5^ cells/well in a 6 well plate. After 24 h, cells were treated with various concentrations of WS (10, 3, and 1 mg/ml) in RPMI 1640 medium with 10% FBS followed by stimulation with LPS (1 μg/ml), and the plate was incubated in the CO_2_ incubator for another 24 h. Cells were lysed using the Trizol reagent and total RNA and cDNA was prepared followed by gene expression analysis as mentioned above. Similar experiments were carried out using A549 cells also. To determine the cytotoxicity of WS, 3-(4,5-dimethylthiazol-2-yl)-2,5-diphenyltetrazolium bromide (MTT) assay was performed. Cells treated as above in the inflammatory profiling setup were treated with MTT (100 μl of 0.5 mg/ml MTT stock in PBS) in an independent experiment, and the plate was incubated for 4 h at 37°C in a CO_2_ incubator. The MTT mixture was aspirated and 100 μl of DMSO was added to each well to dissolve the formazan particles. The plate was shaken for 30 min on a plate shaker at 37°C to ensure complete dissolution of formazan crystals and read at 570 nm to measure the probable cytotoxicity of test substances if any.

Cell viability (%) = Total viable cells (unstained)/Total cells (unstained) + blue stained χ 100.

### LPS-induced inflammatory response in human peripheral blood mononuclear cells (PBMCs)

Intravenous blood was drawn from healthy individuals and collected in a heparinized tube. Heparinized blood was diluted with 1x PBS (1:1). After dilution, blood was layered on Histopaque-^®^1077 (2:1) in a falcon tube and centrifuged at 1,600 rpm for 30 min. The middle layer of the buffy coat containing PBMCs was collected cautiously and washed twice with PBS. The pellet was dissolved in PBS for counting PBMCs using a hemocytometer.

The cells were plated at a cell density of 1 × 10^6^ cells/well in a 24-well plate. Cells were treated with WS (1 mg/ml) and incubated at 37°C in a CO_2_ incubator for 30 min followed by treatment with LPS (1 μg/ml) and incubated again at 37°C in a CO_2_ incubator for another 16 h. Cell culture supernatant was collected the next day and analyzed for the expression levels of different cytokines by ELISA as per the manufacturer’s instructions. Institutional ethics committee approval was obtained prior to initiating experiments with human blood obtained from healthy human volunteers (Institutional ethics approval number: THS1.8.1/100). No experiments were carried out in humans directly.

### LPS-induced neutrophilia in BALB/c mice

The effect of WS in LPS-induced neutrophilia was evaluated under two timepoints. In the first healthy mice were randomized based on their body weights and divided into five different groups: 1) Control; 2) LPS (25 µg/mice); 3) Dexamethasone (1 mg/kg); 4–6) WS, (10, 30, and 100 mg/kg). All the groups except the control group were injected intranasally with LPS in 200 µl PBS (pH 7.4). Mice in groups 4, 5, and 6 were treated orally with WS mixed in saline and challenged with LPS after 30 min of drug treatment. Mice in the control group were challenged with saline only and animals were euthanized 4 h after the WS or saline challenge. Broncho-alveolar lavage (BAL) fluid was collected from the lungs of mice in ice-cold Hank’s Balanced Salt Solution (HBSS) or 1X Phosphate buffer saline (PBS) and processed for the quantification of total and differential leukocyte counts. Lungs were isolated, homogenized, and lysed with Trizol reagent. Total RNA was isolated as per the manufacturer’s protocol. In the second study, the anti-inflammatory potential of WS was analysed in a 4-day LPS challenge model. Mice were treated with WS by oral route followed by intranasal administration of LPS (25 µg/mice) for 4 consecutive days. After 4 days mice were euthanized and analysed for the presence of leukocytes using BAL fluid as in the case of the 4 h study, mentioned above.

### BAL fluid analysis

Smears were prepared using 50–100 µl of the freshly isolated BAL fluid on a frosted glass slide using a Cytospin centrifuge (Biotek, Agilent Technologies Inc. Santa Clara, CA United States) by spinning the slides at 1,000 rpm for 10 min. Slides were dried and fixed with 100% ethanol, further stained with Giemsa stain, and cell counts were taken by scientists who were blinded to the treatment groups.

### Histological evaluation

Healthy mice were randomized based on their body weights and divided into four different groups: 1) Control; 2) LPS (25 µg/mice); 3) Dexamethasone (1 mg/kg); 4) WS (100 mg/kg). All the groups except the control group were injected intranasally with LPS in 200 µl PBS (pH 7.4). Mice were treated orally with WS mixed in saline after 30 min of LPS challenge. Mice in the control group were challenged with saline only and animals were euthanized 4 days after the WS or saline challenge.

Histological evaluation was done using the lung tissues of different groups of mice. Mice were sacrificed 4 days after the LPS administration and lower lobes from the left lung were dissected and fixed in 10% formalin, dehydrated with ethanol followed by embedding in paraffin and cutting into 5 μm sections. After deparaffinization, the tissues were stained with Hematoxylin and Eosin (H&E) as reported previously (Alwahaibi et al., 2015). Pathological changes among different groups were evaluated under a light microscope and lung injury scores were assessed using a semi-quantitative method.

### Statistical analysis

Each experiment was performed three or more times independently with different cell passages. For animal work, each group consisted of *n* = 5 mice. Statistical analysis was performed on GraphPad PRISM 7 software (GraphPad, La Jolla, CA, United States) using one-way ANOVA. The *p*-values of <0.05 were considered statistically significant. The results were calculated as mean ± S.E.M.

## Results

### *Withania somnifera* extract reduces the levels of LPS-induced increase in the levels of pro-inflammatory cytokines in NCI-H460 cells

**FIGURE 1:**
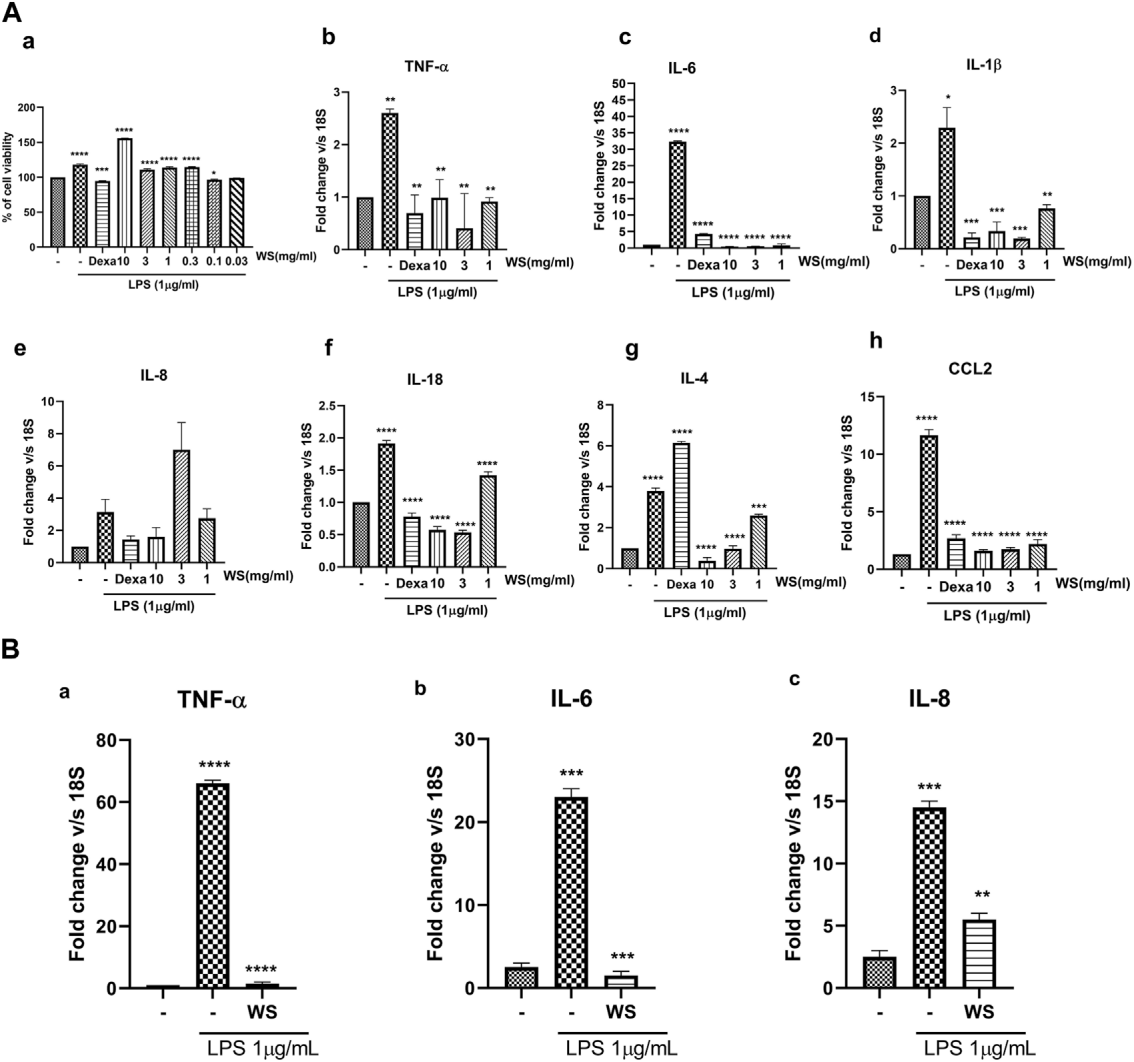
WS suppresses the levels of LPS-induced increase in the mRNA expression of inflammatory cytokines and chemokine(s) *in vitro* in the NCI-H460 and A549 cell lines. **(A)** a) Cytotoxicity of WS on NCI-H460 cells expressed as % cell viability. (b–h) mRNA expression levels of various inflammatory markers were analyzed in the cell lysates by semi-quantitative real-time PCR analysis (TNF-α, IL-6, IL-1β, IL-8, IL-18, IL-4 and CCL2) using NCI-H460 cell line. Cells were pre-treated with WS for 30 min, followed by LPS challenge for 4 h. Dexamethasone was used as a positive control. **(B)** mRNA expression levels of a few key inflammatory markers analyzed by semi-quantitative RTPCR using A549 cell line. Data are presented as the mean ± SEM (*n* = 3). *p < 0.05, **p < 0.001, ***0.0001 ****p < 0.00001 by one-way ANOVA. Significant compared to control (untreated) and ****, ***, ** *significant compared to cells treated with LPS.

To recapitulate the anti-inflammatory properties of WS *in vitro*, cell-based assays were conducted using a lung carcinoma cell line, NCI-H460, and lung epithelial cell line A549. WS extract was not found to be toxic in the MTT assay at the concentrations used in the *in vitro* experiments (Figure 1A). Treatment with LPS led to a significant increase in the secretion of TNF-α, IL-8, IL-6, chemokine (c-c) motif ligand 2 (CCL2), and IL-8 while pre-treatment of the cells with different concentrations of WS (10–0.03 mg/ml) showed a dose-dependent inhibition of pro-inflammatory cytokines by RT-PCR (NCI-H460: Figure 1A) and in A549 cell line for TNF-α, IL-8, IL-6 (Figure 1B) suggesting the anti-inflammatory properties of WS. Treatment with dexamethasone resulted in a decrease in the levels of pro-inflammatory cytokines.

### *Withania somnifera* extract reduces the levels of LPS-induced increase in the levels of pro-inflammatory cytokines in human PBMCs

**FIGURE 2:**
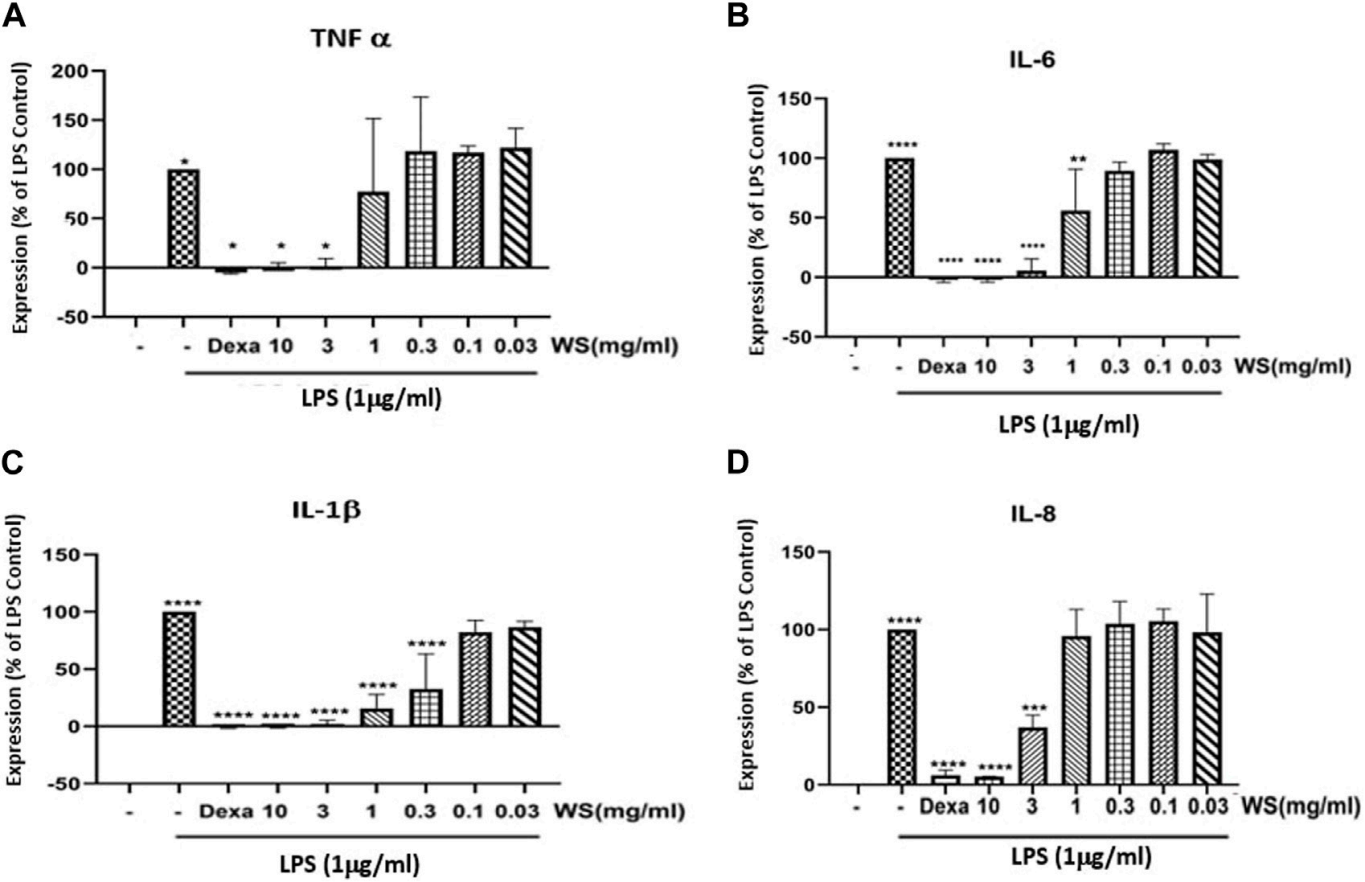
WS inhibits the levels LPS induced increase in the levels of pro-inflammatory cytokines in human PBMCs. PBMC cells were pre-treated with various concentrations of WS for 30 min, followed by treatment with LPS for 24 h. Cell culture supernatant was collected and analyzed for the levels of cytokines and chemokines. **(A)** TNF-α, **(B)** IL-6, **(C)** IL-1β, and **(D)** IL-8 by ELISA. Dexamethasone was used as a positive control. Data are presented as the mean ± SEM (*n* = 3) ****p < 0.00001 by one-way ANOVA. Significant compared to control cells (untreated) and ****, significant compared to cells treated with LPS.

Human PBMCs led to an increase in the expression levels of pro-inflammatory cytokines and chemokines such as TNF-α, IL-6, IL-1β, and IL-8, when induced with LPS (1 μg/ml). Pre-treatment of hPBMCs with different concentrations of WS (10, 3, 1, 0.3, 0.1, and 0.03 mg/ml) on the other hand, significantly reduced LPS-induced increase in the cytokines and chemokines (Figure 2).

### *Withania somnifera (WS)* attenuates the LPS-induced increase in the neutrophil counts in the broncho-alveolar lavage (BAL) fluid of BALB/c mice

**FIGURE 3:**
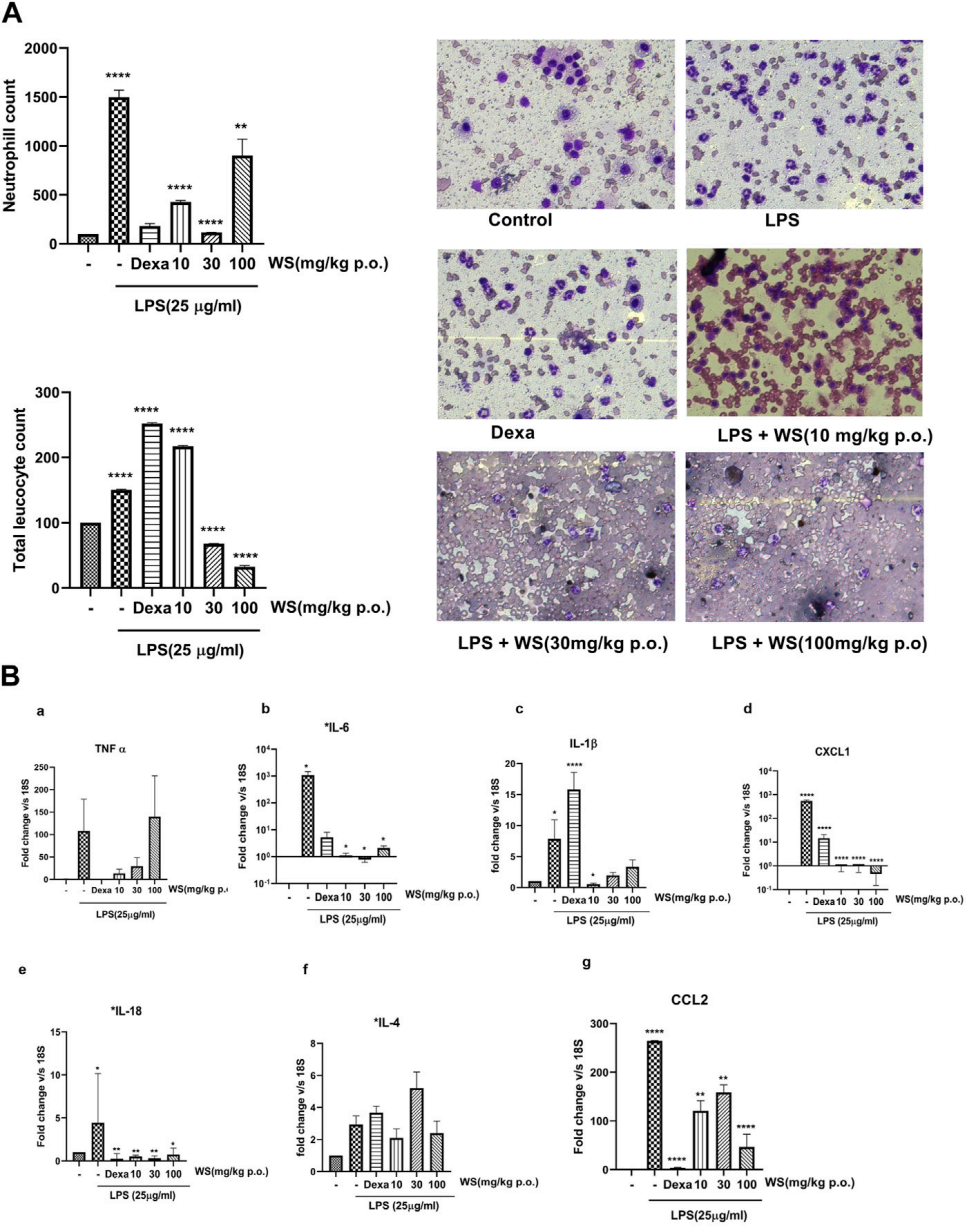
WS shows potent anti-inflammatory potential in BALB/c mice challenged intranasally with LPS for 4 h. **(A)** WS reduced the infiltration of LPS-induced increase in the infiltration of total leukocytes and neutrophils in the broncho-alveolar lavage (BAL) fluid of BALB/c mice. Mice (*n* = 5 mice in each group) were acclimatized, randomized, and prophylactically treated with WS for 30 min followed by LPS challenge for 4 h. Mice were anesthetized after 4 h and BAL fluid was isolated and analysed for a) total neutrophil counts b) total leukocyte counts c) microscopic studies showing the inhibition of neutrophil counts by WS. Data are presented as the mean ± SEM (*n* = 4–5), *p < 0.05, **p < 0.001, ***0.0001 ****p < 0.00001 by one-way ANOVA. Significant compared to control mice and **, **** significant compared to LPS-treated mice. **(B)** WS inhibits the levels LPS induced increase in the mRNA expression levels of pro-inflammatory cytokines in the lung tissue samples of BALB/c mice by real time-PCR. a) TNF-α, b) IL-6, c) IL-1β, d) CXCL-1, e) IL-18, f) IL-4, and g) CCL2. Dexamethasone was used as a positive control. Data are represented as Mean ± SEM, *n* = 5–8, and Statistical significance was assessed by one-way ANOVA. Significant compared to control mice and *, **, **** significance compared to LPS-treated mice *p < 0.05, **p < 0.001, ****p < 0.00001).

To explore the beneficial effects of WS on endotoxin-induced airway inflammation *in vivo*, BALB/c mice were treated with three different doses of WS (10, 30, and 100 mg/kg for 30 min, followed by the LPS challenge by the intranasal route, and BAL fluid was collected after 4 h. Total number of leukocytes and differential neutrophil counts were analyzed in the BALF (Figure 3A). LPS challenge in mice resulted in a significant increase in the leukocytes influx compared to the unchallenged mice. Treatment with different doses of WS attenuated the number of total leukocytes as well as neutrophil counts in a dose-dependent manner. Dexamethasone was used as a positive control and led to a reduction in neutrophil counts.

### *Withania somnifera* reduces the levels of inflammatory cytokines in the lung tissues of BALB/c mice challenged with LPS

LPS-induced leukocyte infiltration is associated with the levels of pro-inflammatory cytokines secreted into the lungs of mice (Gupta V et al., 2015). To test the effect of WS on LPS-induced inflammation in the lung tissue, mRNA expression studies were done using the lung tissues of mice challenged intranasally with LPS with or without WS by RT-PCR. LPS challenge in mice significantly increased the levels of IL-1β, IL-18, IL-4, IL-6, chemokine (c-x-c) motif ligand 1 (CXCL-1), IL-18, CCL2, and TNF-α (Figure 3B) which was found to be significantly reduced in mice pre-treated with WS in a dose-dependent manner. Treatment with dexamethasone resulted in a significant decrease in all tested cytokines except IL-4.

### *Withania somnifera* ameliorates the LPS-induced lung damage in BALB/c mice in a 4-day study

**FIGURE 4:**
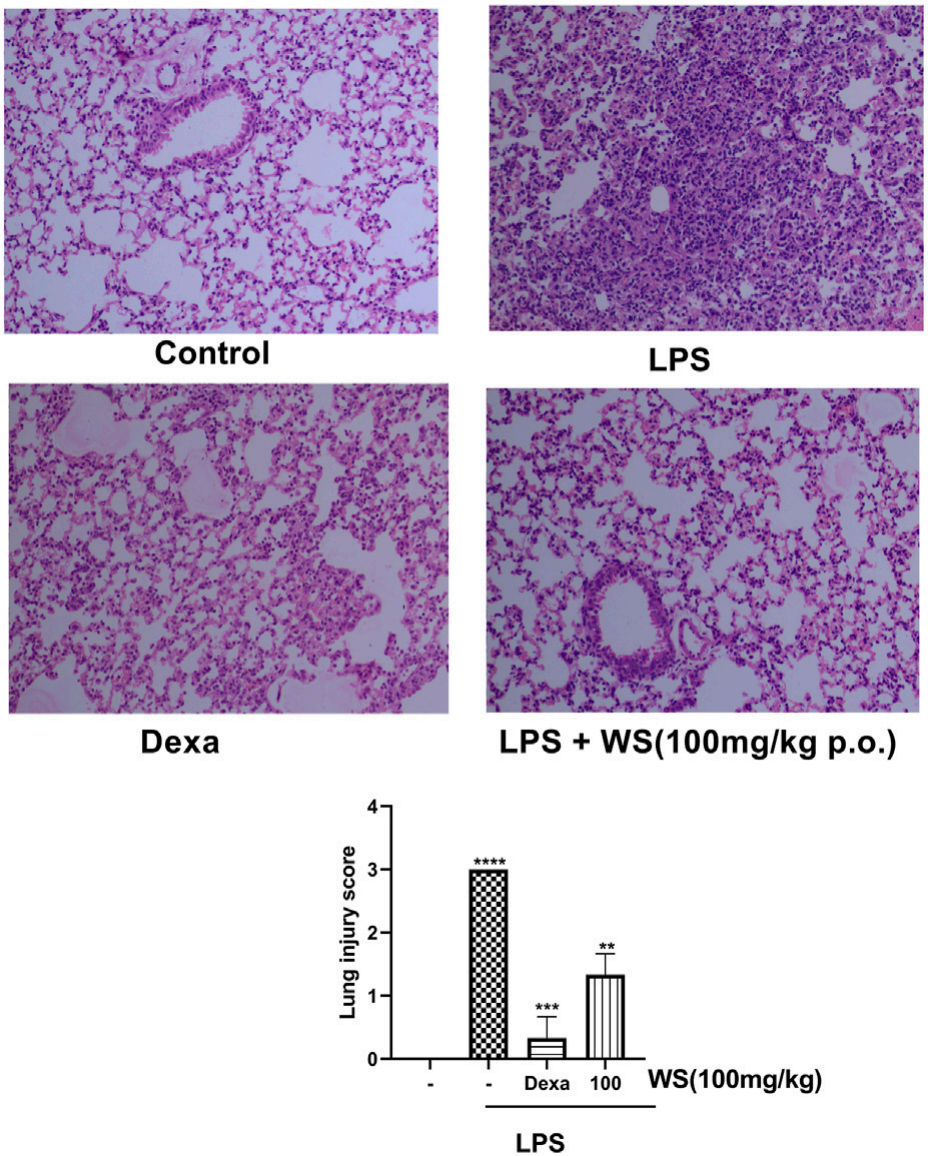
WS ameliorates the LPS-induced lung damage in BALB/c mice in a 4 day study. Effect of WS on LPS-induced lung injury was studied in a 4 day LPS challenge model using BALB/c mice. Lungs from each experimental group ((*n* = 5 mice in each group) were obtained 4 days after LPS challenge as described in the Methodology section. Tissue sections were stained with H&E and sections from each group are represented as control (upper left panel), LPS (1 mg/kg, upper right panel), Dexamethasone (1 mg/kg), lower left panel), LPS+WS (100 mg/kg, lower right panel), H&E stained lung sections are shown in original magnification ×200. Assessment of lung injury was done based on the scores obtained as a mark of lung damage (bar diagram). Scores such as 0, 1, 2, 3, and 4 represented no damage, mild damage, moderate damage, severe damage, and very severe damage, respectively. Data have been expressed as means ± SEM. Statistical significance was assessed using Kruskal-Wallis with a Dunn’s post test, **p < 0.001, **p < 0.0001, ****p < 0.00001). Significant compared to control mice and LPS-treated mice.

Intranasal administration of LPS has been reported to cause lung tissue damage in mice. Histological analysis of the lung tissues was carried out to assess the LPS-induced lung damage in a 4-day LPS challenge model using BALB/c mice (Figure 4). The lungs of mice in the control group, showed normal pulmonary architecture whereas the lungs of mice challenged with LPS showed notable inflammatory cell infiltration and alveolar haemorrhage. Treatment with WS significantly attenuated such pathological changes induced by LPS. The positive control dexamethasone also improved the histopathological conditions in LPS-induced mice. For semi-quantitative evaluation, the changes were also evaluated by calculating a lung injury score our data suggests that pre-treatment WS alleviates LPS-induced pathological changes in the mouse model.

## Discussion

Inflammation is a crucial event for tissue defense against injury or infection. However, uncontrolled or unresolved inflammation can lead to tissue damage or chronic injury with eventual loss of tissue homeostasis. The COVID-19 pandemic in the last 3 years has swept across the globe and has affected a large number of patients with persistent inflammation in vital tissues such as the lungs, metabolic tissues, and the heart. Since aberrant airway injuries are one of the most common complications in these patients, it is important to identify therapies that can alleviate lung inflammation in these patients.

On the basis of the results of randomized clinical trials, WHO has recommended the guidelines for the treatment of COVID-19-infected patients and prescribed the use of corticosteroids to patients who are admitted to hospitals as a result of severe coronavirus infection (WHO, 2022). There is no data however to support the use of systemic corticosteroids in non-hospitalized patients with COVID-19. Since the use of corticosteroids is associated with several adverse events, e.g., hyperglycemia, neuropsychiatric symptoms, and secondary infections in most patients, they should not be prescribed without the controlled supervision of a clinician.

Ayurveda an ancient traditional medicinal system that originated and is being practiced in India illustrates the therapeutic potential of many medicinal plants and herbs in curing various kinds of ailments, diseases, and disorders. *W. somnifera,* popularly known as Ashwagandha, is one such medicinal plant mentioned in Ayurveda and is known for its antiviral, anti-inflammatory, anti-diabetic, neuroprotective, analgesic, anti-tumor, and immunomodulatory properties (Fugner, 1973; Singh et al., 2015; Tiwari et al., 2014) including pulmonary hypertension and fibrosis (Kaur et al., 2015). The anti-inflammatory activity of WS extract and its phytoconstituents has been demonstrated in RAW 264.7 cells by the inhibition of LPS-mediated release of nitric oxide following induction of iNOS and COX-2 expression (Orrù et al., 2023 ) A recently conducted double-blind placebo-controlled clinical trial study (Singh et al., 2022) and recent reviews advocate the benefits of WS extract in the management of COPD patients when given alone or in combination with conventional drugs (Paul et al., 2021; Saggam et al., 2021). A previous study from our lab has shown that WS possesses both antiviral and immunomodulatory properties and was effective in mitigating the pulmonary pathology of COVID-19 in the hamster and transgenic mice models (Rizvi, et al., 2023). In addition, T-cell differentiation was also assessed *in-vitro* to understand the immunomodulatory potential of WS. Effector T helper cell response is central to immunity and health, interferon γ secreted by Th1 cells is important for clearing cellular pathogens (Rizvi, et al., 2023). IL-4 which is primarily secreted by Th2 cells plays a major role in extracellular pathogen clearance and allergic response. Similarly, IL-17 cytokine could act in fungal defense and also leads to cellular injury. We observed that WS was most effective against Th2 cell differentiation suggesting the possible role of WS in limiting COVID-19 pathology. In another study reported recently by us, WS did not have any effect on liver CYP enzymes such as CYP3A4, CYP2C8, and CYP2D6 either alone or in combination with remdesivir suggesting it to be safe without any herb-drug interaction concerns (Kasarla et al., 2022).

Since lung impairment is one of the most common concerns in patients infected with Coronavirus, we, therefore, tested the WS extract for its ability to suppress the inflammation in the cell-based and animal models of acute lung inflammation. The *in vitro* and *in vivo* models used in the present study have wide applicability for testing the anti-inflammatory properties of test substances in alleviating excessive inflammation in respiratory disorders such as asthma, COPD, and bronchitis (Gupta V et al., 2015). The study performed in NCI-H460 and A549 cell lines, demonstrates inhibition of key Th2-dependent cytokines; IL-6 and IL-4, and also a neutrophil function-related cytokine; IL-8 by WS extract in a TLR-4-dependent manner. It is interesting that the level of these cytokines was augmented in COVID-19 patients as well as in the patients with severe asthma and chronic obstructive pulmonary disease (COPD). WS inhibited the expression and generation of the pro-inflammatory cytokines such as IL-6, IL-8, TNF-α, IL-1β, and IL-18 in NCI-H460 cells, human peripheral blood mononuclear cells (PBMCs) and in the lung tissues of Balb/-6 mice challenged intranasally with LPS. Moreover, WS extract pre-treatment reduced neutrophil influx into the LPS-treated mice lungs. Since increased levels of IL-8 have been previously shown to correlate with severe and persistent neutrophil counts in COVID-19 patients, with frequent exacerbations and hospitalizations, we thus evaluated the effect of WS in reducing the LPS-induced neutrophilia in Balb/c mice. Mice challenged intranasally with LPS led to an increase in the total leukocyte and neutrophil counts in the airway which was significantly inhibited by oral administration of WS in both 4-h and 4-day studies along with inhibition of IL-8 in the lung tissues of mice. Ashwagandha thus seems to be efficacious and useful due to its potent immunomodulatory potential.

It is also important to assess the efficacy of Ashwagandha in chronic models of airway inflammation, bronchial asthma in animals (Arora et al., 2021; Arora et al., 2022; Arora et al., 2022; Arora et al., 2022; Visaga et al., 2022), and subsequently in the COPD and COVID-19 patients.

## Data Availability

The original contributions presented in the study are included in the article/supplementary material, further inquiries can be directed to the corresponding authors.
